# Alternative package leaflets improve people’s understanding of drug side effects—A randomized controlled exploratory survey

**DOI:** 10.1371/journal.pone.0203800

**Published:** 2018-09-13

**Authors:** Viktoria Mühlbauer, Roman Prinz, Ingrid Mühlhauser, Odette Wegwarth

**Affiliations:** 1 Health Sciences and Education, MIN Faculty, University of Hamburg, Hamburg, Germany; 2 Harding Center for Risk Literacy, Max Planck Institute for Human Development, Berlin, Germany; 3 Center for Adaptive Rationality, Max Planck Institute for Human Development, Berlin, Germany; Nederlands Instituut voor Onderzoek van de Gezondheidszorg, NETHERLANDS

## Abstract

**Background:**

Current German and EU package leaflets (PLs) do not distinguish to what extent listed side effects are indeed side effects caused by drug intake or instead symptoms that occur regardless of drug use. We recently showed that most health professionals misinterpret the frequencies of listed side effects as solely caused by the drug. The present study investigated whether (1) these misinterpretations also prevail among laypeople and (2) alternative PLs reduce these misinterpretations.

**Methods:**

In March 2017, 397 out of 400 laypeople approached completed an online survey. They were randomized to one of four PL formats: three alternative PLs (drug facts box with/without reading instruction, narrative format with numbers) and one standard PL. Each PL listed four side effects for a fictitious drug: two were presented as occurring more often, one as equally often, and one as less often with drug intake. The alternative formats (interventions) included information on frequencies with and without drug intake and included a statement on the causal relation. The standard PL (control) only included information on frequency ranges with drug intake. Questions were asked on general occurrence and causality of side effects.

**Results:**

Participants randomized to the standard PL were unable to answer questions on causality. For side effects occurring more often (equally; less often) with drug intake, only 1.9% to 2.8% (equally: 1.9%; less often: 1.9%) provided correct responses about the causal nature of side effects, compared to 55.0% to 81.9% (equally: 23.8% to 70.5%; less often: 21.0% to 43.2%) of participants who received alternative PLs. It remains unclear whether one alternative format is superior to the others.

**Conclusion:**

In conclusion, information on the frequency of side effects in current package leaflets is misleading. Comparative presentation of frequencies for side effects with and without drug intake including statements on the causal relation significantly improves understanding.

## Introduction

Informed decision making requires understandable evidence-based health information for patients. Yet studies document that patients are unlikely to receive such information on the Internet or in patient brochures. They are confronted with both a lack of evidence-based decision aids [1–3] and physicians who themselves are often insufficiently informed due to statistical illiteracy [4–9]. This situation may contribute to the findings of a recent survey of the European Medicines Agency, which showed that the majority of patients and healthcare professionals explicitly require greater amounts of unbiased and transparent information on the benefits and harms of medical interventions [10].

When requiring information about a drug’s side effects, up to date package leaflets (PLs) inserted in a drug’s package are the only source of information on drug side effects that patients may definitely receive. However, because PLs have to meet several legal standards, their content rather resembles a legal document than a tool for patient information. At the same time, article 56 of Directive 2001/83/EC of the European Commission requires PLs for medication to be “easily legible” and “clearly comprehensible” [11]. Yet, as the EU commission—on the basis of research from the Netherlands Institute for Health Services Research (NIVEL) and University of Leeds [12]—points out, “patients’ comprehension of the PL and its readability can be improved. The language used is often too complex and the design and lay-out are not always user-friendly. The elderly and those with low literate skills are particularly disadvantaged, but generally these problems hold for all patient groups.”

Not only formal criteria such as readability and layout are insufficiently implemented, however. Current PLs also list “side effects” that occur equally or less often in people taking the drug as compared to people not taking the drug (or placebo in clinical trials). For instance, Barron et al. [13] discovered that 28 of 33 symptoms listed as side effects in PLs for patients with heart failure are not causally related to the intake of beta-blockers. Tan analyzed side effects in official and non-official drug information documents and compared them to the 20 most indicated symptoms in the general population, including back and joint pain, headache, and fatigue. He found that “nine of the 20 symptoms most commonly experienced in daily life are listed as adverse drug reactions in more than half of drug information documents, and 17 are listed by more than a third” [14]. However, German and EU PLs do not contain any placebo group information or reference that the side effects listed are not necessarily caused by drug intake with respective frequencies. The question of causality between drug intake and the occurrence of side effects therefore *cannot* be answered when consulting currently provided PLs. In a recent survey, we showed that even health professionals, who should be particularly skilled in understanding health information, misinterpreted the extent of side effects when presented with a current standard PL [15]. Over 80% of the health professionals erroneously expected a causal relation between the listed frequency of side effects in the PL and drug intake. Their understanding was only slightly improved when provided with a comparative data for a placebo group.

In an attempt to provide physicians with risk information that are easier to approach for their counseling of patients, Barron et al. [13] developed a format that presented the “proportion of symptoms non-pharmacological” next to the “proportion of symptoms caused by the drug,” which corresponds to the proportion of each side effect not attributable or attributable to drug intake. Schwartz et al. had similar intentions when developing the drug facts box, which also displays numerical information on the benefits and harms of medical interventions for both people taking and not taking a drug. The drug facts box has been evaluated with patients and was found to improve understanding, even among those with lower education [16].

Based on these findings and others, a review commissioned by the FDA concluded that quantitative information about benefits and harms presented in terms of absolute numbers as well as in comparison between a drug and a placebo group supports consumers’ understanding of a drug’s efficacy [17]. Claims of benefits of a drug are heavily regulated in PLs, but side effects are not. An increased need for discussing adverse events in PLs was also requested by the NIVEL group [12]. Neither the literature nor stakeholders have reached a consensus on how to best achieve complete and transparent reporting of side effects in spite of this being essential to patients’ right to receive all information required for informed decision making. Not surprisingly, focus group interviews revealed that patients’ most prevailing response to the undifferentiated list of side effects in PLs is fear [18].

The aim of our exploratory study was twofold: First, we wanted to verify whether laypeople would also misinterpret side effects as we previously found health professionals to do. We expected that laypeople would also erroneously assume a causal relation between drug intake and side effects when presented with current forms of German and EU standard PLs. Second, we wanted to investigate whether alternative PLs in comparison to current standard PLs support laypeople in better understanding the causality of side effects due to drug intake.

## Methods

A systematic search of the literature was carried out on PubMed, EMBASE, and PsycINFO [15] using the keywords “patient information leaflet,” “package insert,” “summary of product characteristics,” “direct to consumer advertising,” “instruction leaflet,” “product insert,” “enclosed label,” “drug labeling,” “product labeling,” “adverse effects,” “side effects,” “adverse reactions,” “drug-related side effect,” and respective combinations (PubMed search strategy, see S1 File). To keep the search as sensitive as possible, we did not restrict the keywords by the terms “causal interpretation” or “understanding.” The search was carried out in December 2014 and yielded 5,644 citations. We checked for updates for PubMed and EMBASE on a monthly basis until August 2018. The only inclusion criterion was the examination of understanding the causal relation between symptoms and adverse events in PLs. Fifty-seven articles were read in full text. Up to that time, we could not identify any research on the causal interpretation of adverse events in PLs.

To investigate if alternative PLs potentially enhance people’s understanding of side effects in comparison to currently used standard PLs (control), we decided on three alternative formats (interventions), which have been suggested as a tool for communicating and summarizing findings from medical research: (1) drug facts box according to Schwartz et al. [16], (2) drug facts box according to Schwartz et al. [16] supplemented by reading instructions, and (3) narrative with numbers according to Barron et al. in a modified version [13]. The format of drug facts boxes (format 1)—developed to enhance people’s understanding of medical facts [16]—is a tabular visualization of benefits and harms for a group taking the drug and a group not taking the drug. All event rates for the benefit and harms are provided as absolute numbers, adjusted to the same denominator (e.g., X out of 100), and present the data for the group taking/not taking the drug in columns right next to each other for ease of comparison and to reduce the reader’s cognitive load [16, 19]. As format 2 in our study, we extended the facts box by additionally including reading instructions in order to investigate if it would further supports guidance of people’s interpretation—which to our knowledge has not yet been tested. For the third alternative format, we chose Barron’s et al. [13] approach of narrative with numbers, originally developed (but again not yet tested empirically) for physicians to use in patient communication. Because in its original version a same denominator can refer to different populations and frequencies, and information is presented in percentages and natural frequencies [20, 21], we modified the format by using absolute numbers referring to the same denominator throughout to ensure comparability between the three formats. That is, all three alternative formats included absolute numbers and the same denominators. However, the three formats varied in the extent of how much additional verbal information was given for guidance. Compared to the facts box formats (format 1, format 2) format 3 further did not display the numerical information for the groups taking/not taking the drug in columns next to each other. We decided to include three alternative formats instead of just one in order to explore for the first time if any of these provide a particular advantage over currently used standard PLs in fostering people’s understanding of the causal nature of side effects.

To fill the different formats of a PL with real information, we used data from a systematic review on the side effects of beta-blockers [13]. To exclude the potential bias in response to our questions from people who may know or even take beta-blockers themselves and thus may hold a specific assumption about their side effects, we labeled the provided information on the side effects belonging to a fictitious drug called Suffia. Apart from the drug name, the content of all PLs and the format of the standard PL was not fictitious. Moreover, we took into account that a small survey by Sullivan et al. found that 100 participants were more likely to make direct comparisons between the efficacy of drug-taking and placebo groups when the term “without [drug name]” was used instead of “placebo” or “sugar pill” [22], and accordingly used the term “without Suffia” to describe placebo group results. Format 4 (standard PL) followed the current convention of how information on side effects is presented in the PLs of drugs, which served as the control condition in our study.

For our fictitious drug, we listed four possible side effects: hyperglycemia (“increased blood glucose level”), bradycardia (“slow heart rate”), anemia, and depression. The four side effects were chosen from systematic review on the side effects of beta-blockers [13] by chance, with the only requirement that half of them had a causal relation to drug intake and the other half did not (see S1 Table).

**Fig 1:**
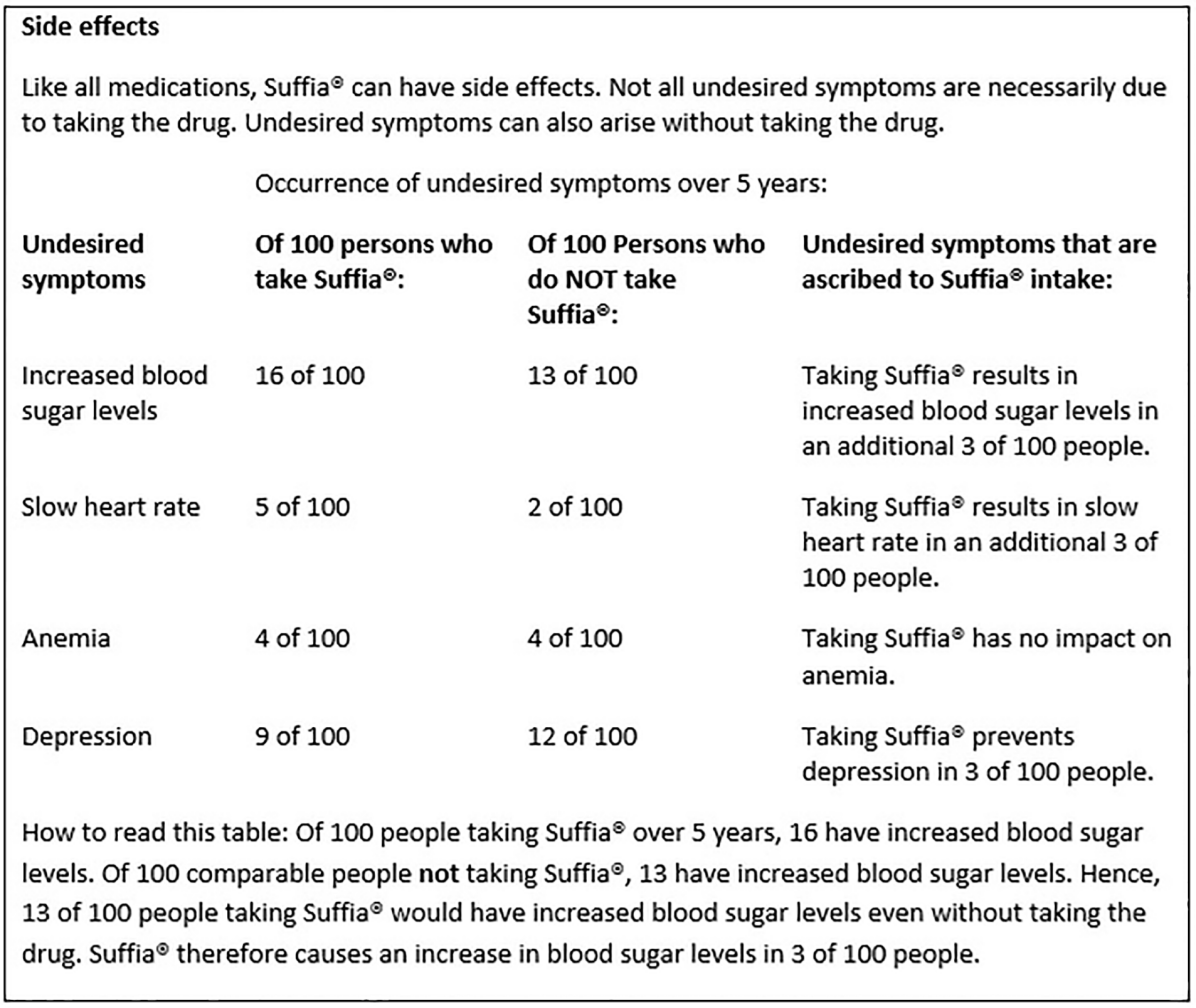
Example of an alternative package leaflet: Drug facts box with reading instructions.

**Fig 2:**
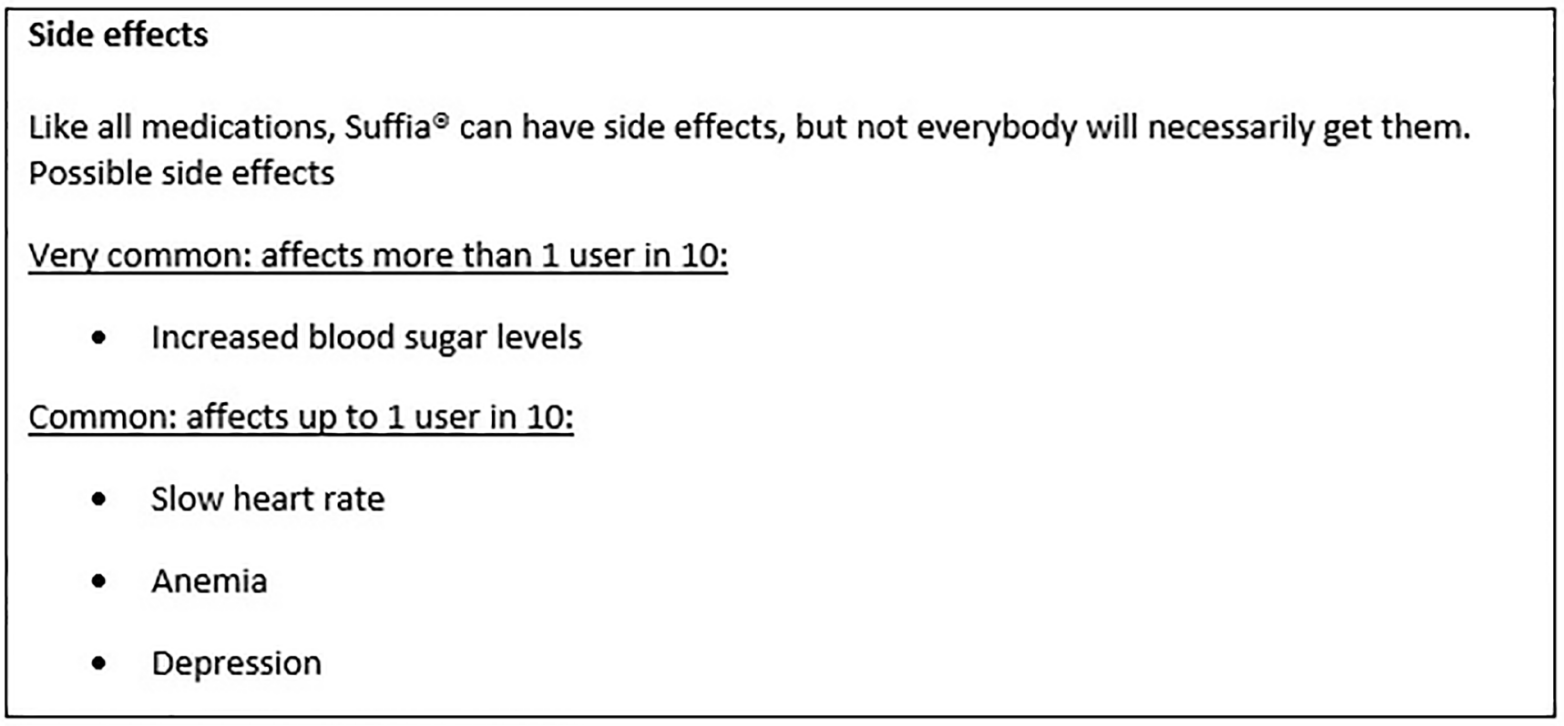
Control: Standard package leaflet.

In the alternative PLs, hyperglycemia (16 of 100 patients who take Suffia vs. 13 of 100 patients who do not take Suffia) and bradycardia (5 of 100 patients who take Suffia vs. 2 of 100 patients who do not take Suffia) were presented as occurring more often in the group taking the drug. Anemia (4 of 100 people who take Suffia vs. 4 of 100 people who do not take Suffia) was depicted as occurring equally often in both groups and depression (9 of 100 patients who take Suffia vs. 12 of 100 patients who do not take Suffia) as occurring less often under drug intake. In each of the alternative formats, a summary statement was additionally given for each of the side effects on how many of these were due to drug intake (e.g., “Taking Suffia leads to 3 extra cases of increased blood glucose levels in 100 people”). We also provided a fictitious time frame within which patients would experience these side effects (“occurrence of undesired symptoms over 5 years”). Fig 1 provides an example of an alternative PL (format 2). For the standard PL (format 4), all information on the side effects was given as in currently distributed PLs and used verbal quantifiers (“very common”, “common”) together with a numerical range (“more than 10 cases in 100 people taking the drug”). That is, hyperglycemia (16 cases out of 100 in the group taking the drug) was labeled as “very common” (“affects more than 1 in 10 patients”), while bradycardia (5 cases out of 100 in the group taking the drug), anemia (4 cases out of 100 in the group taking the drug), and depression (9 cases out of 100 in the group taking the drug) were labeled as “common” (“affects up to 1 in 10 patients”) (see Fig 2). The remaining PLs can be seen in the Supporting Information (S1–S8 Figs).

The online version survey was set up and published using Limesurvey, an open source survey software (Limesurvey GmbH, Hamburg, Germany; URL: http://www.limesurvey.org). Consent was recorded by participants ticking the box agreement of participation after reading information about the research goals and the data collection (consent form and introduction to the Online Survey, see S2 File). If participants did not agree to participate, they automatically exited the survey. Data were collected on 22 March 2017 using clickworker, an online survey panel provider (clickworker GmbH, Essen, Germany; URL: http://www.clickworker.de). Participants received the average reimbursement of 1 euro—as suggested by clickworker—for their time spent on participating in our survey. The study was conducted in German; all formats and survey questions in this publication are translations. Original phrasing of the PLs can be seen in the Supporting Information. Of the 400 participants who entered the survey, 397 provided their informed consent and were randomly assigned to one of the four formats of the PL. Randomization was achieved by means of a computer-based random number generation and neither participants nor investigators could foresee the random assignment. After reading the respective PL, participants were asked for each of the listed side effects 1) how many people out of 100 taking Suffia^®^ would experience the respective side effects (general occurrence), and 2), for how many people out of 100 taking Suffia^®^ the respective side effect is causally induced by the drug (causality). The order of these questions was randomized across participants. To best reflect a real-life situation, the respective PL remained visible the entire time while participants were answering the question. That is, participants were not required to recall the numbers of people experiencing side effects with and without drug intake when making their judgments.

1. General Occurrence Question:
  “Out of 100 people taking the drug Suffia^®^: How many people experience the following side effects during intake of Suffia^®^?”
  [Original language: Von 100 Personen, die das Arzneimittel Suffia^®^ einnehmen: Bei wie vielen Personen treten die folgenden Nebenwirkungen unter der Einnahme von Suffia^®^ auf?]
2. Causality Question:
  “Out of 100 people taking the drug Suffia^®^: For how many people is taking Suffia^®^ the cause of experiencing the following side effects?”
  [Original language: Von 100 Personen, die das Arzneimittel Suffia^®^ einnehmen: Bei wie vielen Personen ist das Einnehmen von Suffia^®^ ursächlich für die folgenden Nebenwirkungen?]

The general occurrence question tested whether participants were able to derive correct numbers from the table. The correct answer was the number of people experiencing the symptom among those taking the drug. The question regarding causality investigated whether the respective PLs enable people to correctly understand to what extent side effects listed in the PL are causally induced by drug intake. Here the correct answer was the difference in the event rate between the group taking and not talking the drug.

The survey did not allow for item nonresponse; thus all 397 surveys were complete. For all formats of the PL, answers to the general occurrence question were rated correct according to the aforementioned frequencies. For the question on causality, the correct answers were “3 out of 100 people” for hyperglycemia and bradycardia and “0 out of 100 people” for anemia and depression for all formats of the PL.

### Analysis

Taking into consideration our first study with health professionals [15] and the results of a randomized controlled trial on the effectiveness of a drug facts box on people’s understanding by Schwartz and Woloshin [23], we assumed a difference of at least 20 percent points between the standard PL and all alternative formats in the proportions of correct responses to the causality question (question 2). However, due to a lack of previous studies comparing differences in effectiveness between the three alternative formats we used in the study at hand, we were not able to estimate the potential differences between the alternative formats or to estimate the potential size of differences in supporting people’s understanding of medical facts. Thus, except for the difference between the standard PL and alternative PLs, all other analyses were only hypothesis generating.

Data were analyzed by frequency, reporting for each group and side effect. Order effects were analyzed using the non-parametric Pearson Chi-square test. All data were stored and analyzed with IBM SPSS Statistics 24 (New York City, USA) and RStudio (RStudio Inc, Boston, USA). Graphics were produced with the RStudio package ggplot2 by H. Wickham (2009). Because analyses were not pre-defined but hypotheses generating we did not adjust for multiple testing or baseline imbalances between groups. Statistical analysis was not blinded.

### Ethical approval

The study was approved by the Institutional Ethics Board of the Max Planck Institute for Human Development, Berlin (Germany).

## Results

Age of participants in the study ranged from 18 to 69 years (*Mean* = 37.0, *SD* 12.0). Mean time spent on reading the respective format and answering both questions was 3 min. 11 sec. (*median* = 2 min. 29 sec.; *SD* = 3 min. 57 sec.). See Table 1 for participants’ characteristics.

**Table 1:** Characteristics of survey participants.

	Group 1 (n = 91) Drug facts box (format 1)	Group 2 (n = 95) Drug facts box with instruction (format 2)	Group 3 (n = 105) Narrative with numbers (format 3)	Group 4 (n = 106) Standard package leaflet (format 4)
**Mean age** (SD); (range)	36.9 (11.6); 18–65	38.0 (12.3); 18–69	37.1 (11.7); 18–63	36.4 (12.7); 18–65
**No (%) female**	40 (44.0)	51 (53.7)	54 (51.4)	43 (40.0)
**No (%) speaking German at home**	89 (97.8)	91 (95.8)	98 (93.3)	102 (97.1)
**No (%) education**				
Secondary (8 years)	4 (4.4)	1 (1.1)	3 (2.9)	3 (2.9)
Secondary (10 years)	22 (24.2)	21 (22.1)	20 (19.0)	40 (38.1)
University entrance diploma (12–13 years)	65 (71.4)	73 (76.8)	82 (78.1)	62 (59.0)
**No (%) occupational status**				
Untrained	12 (13.2)	12 (12.6)	14 (13.3)	14 (13.3)
Vocational training	33 (36.3)	24 (25.3)	29 (27.6)	47 (44.8)
Professional school	8 (8.8)	9 (9.5)	3 (2.9)	10 (9.5)
University of applied science	3 (3.3)	14 (14.7)	17 (16.2)	6 (5.7)
University graduate	34 (37.4)	36 (37.9)	39 (37.1)	26 (24.8)
Other	1 (1.1)	0	3 (2.9)	2 (2.0)
**No (%) currently employed**	51 (56.1)	63 (66.3)	78 (74.3)	74 (70.5)
**No (%) health professional**	2 (2.2)	3 (3.2)	2 (1.9)	2 (1.9)

### a) General occurrence of side effects during drug use

Asked about the number of people who experience each of the side effects listed in the PL during intake of the drug Suffia^®^, between 68.1% and 75.8% of the participants presented with the drug facts box (format 1) gave a correct answer across the questions on the four listed side effects. For those presented with the drug facts box with reading instruction (format 2), between 52.6% and 55.8% responded correctly. Among people presented with the narrative including numbers (format 3), between 55.2% and 93.3% derived the correct number from the table. In contrast, participants receiving the standard PL (format 4) gave considerably fewer correct answers to the questions on general occurrence. Only between 0% and 11.3% of these participants were able to provide correct answers across the four questions. Detailed information on correct responses per format and listed side effects can be seen in Table 2.

**Table 2:** Participants’ correct responses to the question on the general occurrence of side effects per format and per side effect.

	Drug facts box (n = 91)	Drug facts box with reading instruction (n = 95)	Narrative with numbers (n = 105)	Standard package leaflet (n = 106)
	Percentage of participants with correct responses [95% CI]			
**Hyperglycemia**	68.1 [57.9, 76.8]	52.6 [42.7, 62.4]	55.2 [45.7, 64.4]	0.0 [0.0, 3.5]
**Bradycardia**	68.1 [57.9, 76.8]	53.7 [43.7, 63.4]	56.2 [46.7, 65.3]	6.6 [3.2, 13.0]
**Anemia**	75.8 [66.0, 83.4]	55.8 [45.8, 65.4]	93.3 [86.9, 96.7]	1.9 [0.5, 6.6]
**Depression**	71.4 [61.4, 79.7]	53.7 [43.7, 63.4]	61.9 [52.4, 70.6]	11.3 [6.6, 18.8]

**Fig 3:**
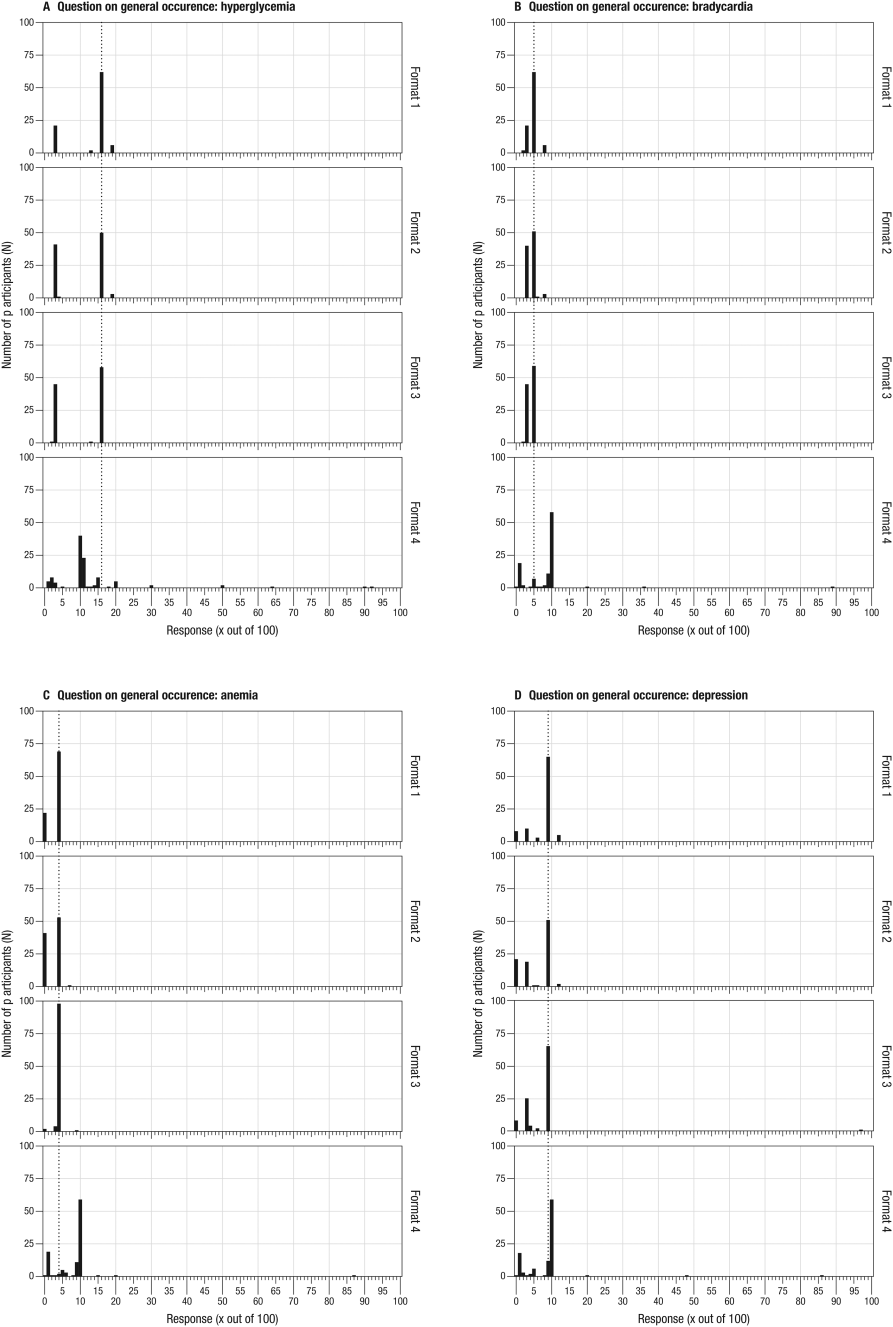
Participants’ response distribution on the general occurrence of side effects per format. (Format 1 = drug facts box, format 2 = drug facts box with reading instruction, format 3 = narrative with numbers, format 4 = standard PL).

The formats also resulted in different variances of participant’s responses on the general occurrence of side effects. Participants receiving the standard PL (format 4) tended to have larger variance than did participants who received the alternative formats 1 to 3. For instance, for hyperglycemia (correct answer: 16), the responses of participants receiving the standard PL ranged from 1 to 92 out of 100 people with a median of 10 (*M*_*responses*_ = 13.1; *SD* = 14.1) (Fig 3). The majority of participants who received the standard PL underestimated the general occurrence of side effects: 87.7% provided frequencies lower than the correct answer of 16.

Although the majority of people receiving any of the alternative formats provided the correct answer regarding the general occurrence, a considerable number of participants provided the response that would have been correct for the question on the causality of side effects (correct response for hyperglycemia: 3). That number was provided by 23.1% of participants receiving the drug facts box (format 1) (range _*responses*_: 3–19, *M*_*responses*_ = 13.1; *SD* = 5.7), 43.2% of participants receiving the drug facts box with reading instruction (format 2) (range _*responses*_: 3–19, *M*_*responses*_ = 10.4; *SD* = 6.6), and 42.9% of those receiving narratives with numbers (format 3) (range _*responses*_: 2–16, *M*_*responses*_ = 10.3; *SD* = 6.5). A similar pattern was observed for responses on the question for bradycardia, but not for anemia and depression. Information on the range, mean, and standard deviation of responses for each of the other three side effects can be found in the Supporting Information (see S2 Table).

As mentioned before, the general occurrence question aimed at investigating whether participants were able to correctly draw numbers from a table. Because participants receiving the standard PL had to deal with changing denominators and imprecise numerical information (i.e., ranges instead of point estimates), they were generally at a disadvantage in arriving at a correct response to this question. If the responses to the general occurrence question are analyzed more liberally for this group, allowing for a ballpark of +/-5 out of 100, between 38.7% and 97.2% answered the question correctly.

### b) Causality between drug use and side effects

Asked about how often each of the side effects listed in the PL is causally induced by the intake of the drug Suffia^®^, between 29.7% and 57.1% of people presented with the drug facts box (format 1) were able to provide the correct answers (see Table 3). Of the people presented with the drug facts box with reading instruction (format 2) between 43.2% and 70.5% responded correctly, and among people being presented with the narrative including numbers (format 3), between 21.0% and 81.9% provided correct estimates. Interpretation of the causal extent of the side effect depression—depicted as occurring less frequently in the group taking the drug—appeared challenging, however. Whereas over 50% of participants presented with any of the three alternative formats correctly understood the causal relation between drug intake and each of the other side effects, less than 50% did so for depression (see Table 3). Also, the alternative formats did not prevent some participants from confusing causality with general occurrence. For instance, for hyperglycemia, between 15.2% and 37.4% of the participants provided the correct response for general occurrence instead of causality. For the other side effects, see Supporting Information.

**Table 3:** Participants with correct responses to the question on causality per format and per side effect.

	Drug facts box (n = 91)	Drug facts box with reading instruction (n = 95)	Narrative with numbers (n = 105)	Standard package leaflet (n = 106)
	Percentage of participants with correct responses [95% CI]			
**Hyperglycemia**	55.0 [44.7, 64.8]	69.5 [59.6, 77.8]	81.9 [73.5, 88.1]	2.8 [1.0, 8.7]
**Bradycardia**	55.0 [44.8, 64.8]	68.4 [58.5, 76.9]	80.0 [71.4, 86.5]	1.9 [0.5, 6.6]
**Anemia**	57.1 [46.9, 66.8]	70.5 [60.7, 78.8]	23.8 [16.7, 32.8]	1.9 [0.5, 6.6]
**Depression**	29.7 [21.3, 39.7]	43.2 [33.7, 53.2]	21.0 [14.3, 29.7]	1.9 [0.5, 6.6]

With the standard PL (format 4), a minority of participants correctly understood the extent of the causal relation between side effects and drug intake: Only between 1.9% and 2.8% estimated the correct answers to the respective questions. Table 3 shows the results for each format of the PL and each side effect in detail.

**Fig 4:**
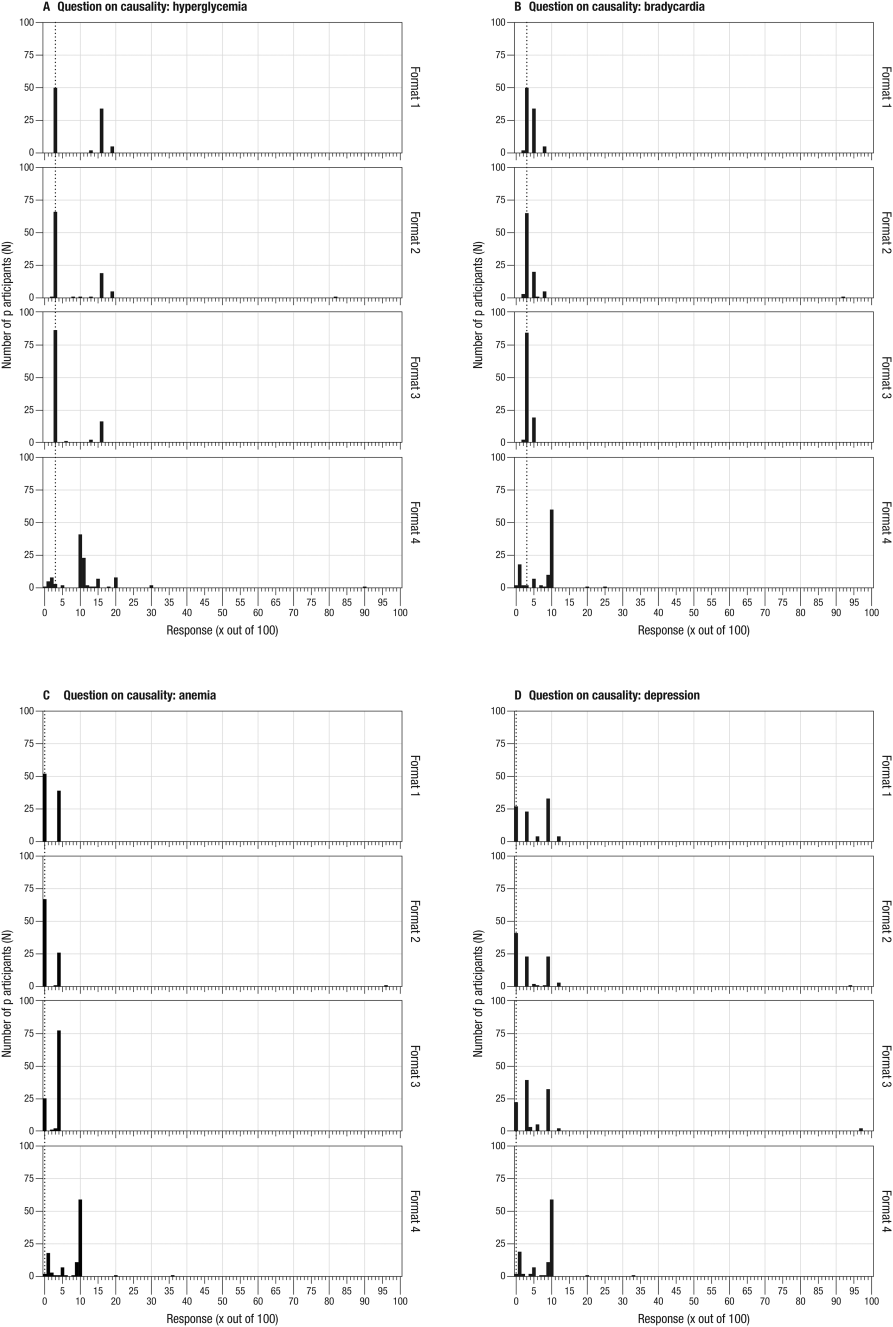
Participants’ response distribution on the causal extent of side effects per format. (Format 1 = drug facts box, format 2 = drug facts box with reading instruction, format 3 = narrative with numbers, format 4 = standard PL).

The formats again resulted in different variances of participants’ responses. Participants receiving the standard PL (format 4) again tended to display a larger variance than participants who received the alternative formats 1 to 3. For instance, taking participants’ estimates of the causal extent of hyperglycemia due to drug intake (correct answer: 3), participants receiving the standard PL provided responses ranging from 0 to 90 (*M*_*responses*_ = 11.2; *SD* = 9.4, *median*_*responses*_ = 10) (see Fig 4). Now a majority of participants in this group overestimated the extent of side effects causally induced by the drug intake: 84.0% provided frequencies higher than the correct answer of 3. For the alternative formats, responses on the causal extent of hyperglycemia by participants receiving the drug facts box (format 1) ranged from 3–19 (*M*_*responses*_ = 9.0; *SD* = 6.7), by participants receiving the drug facts box with reading instruction from 2–81 (*M*_*responses*_ = 7.5; *SD* = 9.7, median _*responses*_: 3.0), and by participants receiving narratives including numbers (format 3) from 3–16 (*M*_*responses*_ = 5.2; *SD* = 4.8), respectively (Fig 4). Information on the range, mean, and standard deviation of responses for each of the other three side effects can be found in the Supporting Information (see S3 Table).

Randomization of the questions on general occurrence and causality did not influence the proportion of correct response rates (results for order effects, see S4 and S5 Tables), except for the drug facts box (format 1) and the question on depression (*p* = 0.04), where participants who first viewed the question on causality provided more correct replies to the general occurrence question afterwards.

## Discussion

In our study, we showed that currently used standard formats of PLs do not enable laypeople to distinguish which proportion of listed side effects are indeed side effects caused by drug intake or instead are symptoms occurring regardless of drug intake. Nearly all participants in the group receiving the standard PL were unable to draw correct conclusions on the causal extent of side effects and overestimated the frequency of all listed side effects. In contrast, all alternative formats improved understanding of the extent to which these side effects are causally induced by the drug. Particularly for the side effects presented as occurring more often in the group of people taking the drug as compared to not taking the drug, the majority of participants were able to correctly understand the causal link between drug intake and side effects when presented with the alternative format. Narratives with numbers and drug facts boxes with reading instructions might be particularly helpful in this context. Although the alternative formats, in contrast to the standard format, improved people’s understanding of the causality of all side effects, these formats could only partly prevent the misunderstanding of side effects that were depicted as occurring equally or less often under drug intake (anemia and depression). We assume that the word “side effect” triggers a certain anticipation. To find a listed side effect occurring less often within people taking a drug than within those not taking the drug may contradict the conventional wisdom of what “side effect” means. More neutral descriptions such as “unintended events” may have prevented some of the confusion we found for the question on the causal extent of depression due to drug intake. However, because “side effects” is the official term, well-known and commonly used, we decided to use it in our study. We can only speculate, however, why, among the three alternative formats, people receiving the narrative with numbers (format 3) particularly suffered from the aforementioned effect. In contrast to the facts box formats (formats 1 and 2), which displayed outcome information for the intervention and control group side-by-side, within the format “narrative with numbers” numbers had to be extracted from sentences, which might have contributed to the variability of our findings. Considering the lack of power for comparability between the alternative formats, variability might also be purely due to chance. We further found an order effect for the facts box (format 1) and depression, where participants who first viewed the question on causality provided more correct replies to the general occurrence question afterward. The order effect just gained significance and the respective effect size (0.22) was small. As can be seen in the S2 Table in the Supporting Information, for the other three side effects we also saw a trend toward the same order effect for the facts box format. Although again speculative, the reason for this finding might be that, in contrast to the other two alternative formats, the facts box in its pure form does not provide any additional guidance by reading instructions or verbalizations of the findings next to the provision of the numbers. Studying the effect of people’s interpretation on the causality of “side effects” occurring equally or less often under drug intake by using a different terminology for describing “side effects,” and further using larger sample sizes for studying differences in the interpretation on the nature of side effects caused by the alternative PLs within future research, will likely help shed more light on the underlying mechanisms.

PLs have been in the focus of various research activities. Particular attention has been paid, for example, to the wording used in PLs [24] or to which layout (font size, color, bold print) best supports the consumer’s ability to find and understand important information contained in PLs [25]. Our study adds to the existing body of research in that it is the first to show that alternative formats of PLs, such as facts boxes, in comparison to currently used standard PLs, considerably improve laypeople’s understanding of the proportion of side effects that are causally related to drug use. The study was sufficiently powered to detect differences between currently used standard and alternative PLs. Further, to the best of our knowledge, the study is also the first to test the format “narratives with numbers”—originally designed by Barron et al. [13]—with laypeople, although in a modified form.

Patient empowerment has undoubtedly taken a huge step forward that pharmaceutical companies are required to provide PLs with data on side effects. Our research should therefore not be misunderstood as a proposal to include only side effects with proven causal relations. Retrieving reliable data on the causality of side effects that occur very rarely (<1/1000) is often impossible. It would require an enormous number of participants to determine such rare events in clinical trials, which is why these are usually identified by post-marketing surveillance activities. However, informed decision making would already be greatly enhanced if information on causality for side effects occurring in 1% or more people were included in PLs. Already today the FDA recommends that symptoms for which solid evidence has shown that these are not caused by the drug should not be included in the PL. It might also be worthwhile to present different adverse events sections in the PL: one for those supported by high quality evidence and one for other adverse events. Way et al. [26] conducted a survey asking patients with chronic conditions and the general public whether they desired medical information that had not undergone scientific analysis or if they preferred to wait until safety information had been further investigated. 51% of the patient group and 63% of the general public said they preferred receiving information on potential side effects as soon as there is a sign of a safety problem. These findings may support the idea of including different sections that distinguish between already proven side effects and adverse events still under investigation.

As our study documents, current standard PLs lead to a high degree of misperception about side effects and hence pose a considerable threat to informed choices. As findings from focus group interviews revealed, people fear taking drugs after reading currently used PLs [18], what may increase the risk for not adhering to their medication [27]. Further, verbal expressions like “very common” or “common”—consistently used in current standard PLs—influence expectations of the occurrence of side effects even in combination with frequency expressions, for instance, among people from ethnic minorities, people with less education, or people with negative beliefs about medicines [28]. Expectations from verbal suggestions are found to trigger the nocebo effects [29]. Alternative PLs may potentially help to reduce fear and foster informed choices.

Listing symptoms that are “prevented” by drug intake should not, however, result in a form of hidden advertising, as has occurred for some drugs. For instance, during the German benefit assessment of pharmaceuticals in accordance with the German Social Code, Book Five (SGB V), for the antidiabetic drug dapagliflozin, positive side effects such as weight loss and lower blood pressure were strongly emphasized [30] while long-term data on cardiovascular outcomes were missing.

The fact that current PLs do not allow conclusions to be drawn on the causality between drug intake and the frequency of side effects has further consequences on physicians’ and other health professionals’ workload. Lacking solid information on drugs’ effectiveness in current PLs, physicians dedicated to initiating informed decision making in their patients are forced to search the scientific literature themselves to identify the extent of side effects caused by the specific drug. But many databases for adverse events are invalid because of nonsystematic assessment [31] and expectations (from investigators and patients) in clinical trials [32], incomplete reporting, missing transparency of clinical trial results, and spontaneous reporting after market entry. Köhler et al. [33] compared the proportion of adverse events of new drugs reported in various publication types. Whereas journal publications report 40% of all adverse events, registry reports contain 53% and EPAR (European public assessment reports) only 20%. Reporting of serious or drug-specific adverse events was even more scarce.

The practical consequence of the present study is to substantially increase regulatory efforts to change the presentation of numerical information on the occurrence of side effects in PL in the best interest of patients. However, current developments on this issue are not encouraging. Despite increasing evidence that alternative forms as compared to the current form of presenting medical risk information can improve patients’ understanding, the FDA does not see the need for new regulations. Their reasoning is “that the inclusion of quantitative information about the risks and benefits of prescription drugs in a single standardized format would not broadly improve health care decision making” [17]. The EMA recently announced work on improving the PL to better meet the need of patients and healthcare professionals [10]. Yet, they do not specify how they will go about this task and how changes should be implemented. From an ethical and patient-centered perspective, these objections are hard to understand. Risking that patients refrain from taking beneficial drugs after overestimating side effects is unjustifiable. Our data clearly demonstrate the high degree of misunderstanding induced by current PLs and that it is possible to improve the PL format and, consequently, patients’ understanding. PLs urgently need to be refined in the interest of informed decision making. It is now up to regulatory agencies to take action.

### Limitations

Our study needs to be viewed in the light of limitations. First, the study was neither designed nor powered to detect differences between the three alternative formats. However, power calculation was not possible because previous research providing sufficient information on what differences to expect between these aforementioned conditions was lacking. Given that each of the three alternative approaches used in our study has been suggested to improve people’s understanding of medical research [16, 19], we nevertheless believe that our exploratory approach to study the relative effectiveness of each of the three alternative format in comparison to the current standard PL in just on study is justified. Whereas the present study therefore cannot ascertain whether one of the three alternative formats is superior to the others on communicating side effects occurring with different frequencies, it nevertheless provides first insights on the relative effectiveness of these different alternative approaches in communicating side effects over the currently used approach. Second, we did not test the wording of the questions used in our study in previous focus groups. A certain number of participants misinterpreted the general occurrence question as a question on causality. The format of an online survey makes it impossible to identify whether this occurred due to the wording of the question in the survey or because the effect of presenting a comparison between people taking or not taking the drug already strongly implies causality that asking for anything beyond the true (causal) extent of side effects did not seem reasonable. Asking both questions concurrently might have made it easier for the participants to detect the different intentions behind both questions. Third, compared to the general population, participants in our survey were educated above average and younger. Our sample is therefore not representative, but we can rule out bias due to a health professional background. Further, participants who were randomized to the standard PL differed in their levels of education compared to the other groups. However, because the proportion of correct responses for that format differed so substantially from all three alternative formats, we are confident that our main findings are primarily explained by the way information is presented in the different PLs rather than by the difference in education. Fourth, we used data from placebo groups to illustrate how often different symptoms occur without drug use. Placebo group data are the best available surrogate for the occurrence of symptoms without drug intake. However, adverse events reported under placebo can sometimes be similar to those expected for active treatment [32]. The fact that participants in clinical trials are aware of being in a trial, receiving special attention and a form of “treatment”—be it an active drug or a placebo—can lead to positive (placebo) or negative (nocebo) symptoms that might not occur under daily life conditions. Therefore, all values presented in our PLs are only rough estimates, without acknowledging placebo and nocebo effects. It is an unsolved problem that data without placebo or nocebo effects cannot be obtained. However, we refrained from describing the drug as a placebo or sugar pill in our PLs to keep the text easily understandable for laypeople, and to acknowledge the fact that our previous research with health professionals found only minor improvements in understanding when providing a placebo column without any additional explanation [15]. Fifth, estimating the number of adverse events caused by drug intake through calculating the difference between people experiencing the side effect while taking the drug and those experiencing these events while taking a placebo might seem too simplistic. In contrast to people participating in RCTs, those taking the drug in “real life” are often prone to multimorbidity and polypharmacy and might experience deviating rates. However, the aim of our study was not to establish evidence on the true size of side effects under real circumstances, but to investigate which format would likely support people’s understanding of side effects. Sixth, we presented only four adverse events within the PLs of our study in order to keep time load for working through the survey acceptable and ensure sufficient participation rates. Tan et al. [14] analyzed information on side effects for 15 prescription drugs in PLs and other sources and found that the median of side effects listed in these documents ranged from 26 to 74.5. Further research needs to assess whether alternative PLs still improve understanding when they contain a larger number of side effects than depicted in our study.

## Conclusion

Laypeople commonly assume a causal relation between drug intake and the frequency of side effects when reading current PLs. With these PLs, it is impossible to find information on the causality of side effects induced by drug intake. Our study showed that a considerable number of people confronted with standard PL overestimate the extent of side effects. Considering that these PLs may leave a considerable number of people in fear and potentially affect their adherence to prescribed drugs, the current situation not only undermines informed decision making but asks for a profound change in how information is given in package leaflets. Our study demonstrated that alternative formats exist that would help people to better understand information on side effects. Yet most people are probably not even aware of the fact that substantial information is lacking in current PLs. Including all relevant information on side effects for both intervention and control group might require more space than available on current standard PLs; yet, we believe that thinking of new layout formats is more justified than withholding relevant information from patients. We now need the commitment of regulatory instances to change existing standard PLs and include comparative information on people taking and not taking the drug, in the best interest of patients and of health care.

## Supporting information

S1 File PubMed search strategy. (PDF)

S2 File Consent form and introduction to the survey. (PDF)

S1 Fig Format 1: Alternative package leaflet (intervention): Drug facts box. (PDF)

S2 Fig Format 2: Alternative package leaflet (intervention): Drug facts box with reading instruction. (PDF)

S3 Fig Format 3: Alternative package leaflet (intervention): Narrative with numbers. (PDF)

S4 Fig Format 4: Standard package leaflet (Control). (PDF)

S5 Fig Format 1: Drug facts box (Original language). (PDF)

S6 Fig Format 2: Drug facts box with reading instruction (Original language). (PDF)

S7 Fig Format 3: Narratives with numbers (Original language). (PDF)

S8 Fig Format 4: Standard package leaflet (Original language). (PDF)

S1 Table Excerpt from Barron et al.: The proportion of side-effects on beta-blocker that are caused by being on beta-blocker. (PDF)

S2 Table Distribution of participants’ responses on the general occurrence of side effects during drug intake for each format. (PDF)

S3 Table Distribution of participants’ responses on the causal relation between drug intake and side effects for each format. (PDF)

S4 Table Effect of the order of the question of general occurrence on the proportion of participants’ correct responses in dependence on whether participants first received the question on general. (PDF)

S5 Table Effect of the order of the question of causality on the proportion of participants’ correct responses in dependence on whether participants first received the question on causality and then the question on general occurrence or vice versa occurrence and then the question on causality or vice versa. (PDF)
