# Supplementary material for: Alternative package leaflets improve people’s understanding of drug side effects—A randomized controlled exploratory survey
Source: PLoS One. 2018 Sep 13;13(9):e0203800. doi: 10.1371/journal.pone.0203800 (PMC6136776; doi:10.1371/journal.pone.0203800)
Supplement: S1 File — (PDF) [file pone.0203800.s001.pdf]

S1 File. PubMed search strategy

((("patient information leaflet" OR "patient information leaflets" OR "package insert" OR "package inserts" OR "summary of product characteristics" OR "dtc advertising" OR "direct to consumer advertising" OR "instruction leaflet" OR "instruction leaflets" OR "product insert" OR "product inserts" OR "enclosed label" OR "drug labeling" OR "product labeling") OR ("Drug Labeling"[Mesh] OR "Product Labeling"[Mesh]))) AND (("adverse effect" OR "adverse effects" OR "side effect" OR "side effects" OR "adverse reaction" OR "adverse reactions" OR "drug related side effect" OR "drug related side effects") OR ("Drug-Related Side Effects and Adverse Reactions"[Mesh]))
