# Supplementary material for: Alternative package leaflets improve people’s understanding of drug side effects—A randomized controlled exploratory survey
Source: PLoS One. 2018 Sep 13;13(9):e0203800. doi: 10.1371/journal.pone.0203800 (PMC6136776; doi:10.1371/journal.pone.0203800)
Supplement: S2 File — (PDF) [file pone.0203800.s002.pdf]

S2 File. Consent form & Introduction to the survey

Max-Planck-Institut für Bildungsforschung
Max Planck Institute for Human Development

Harding Center for Risk Literacy
Center for Adaptive Rationality

Declaration of Consent for Majority-Aged Participants

In the study “Do alternative package leaflets enhance people’s understanding of side effects as compared to currently used standard package leaflets” we intend to examine how people draw conclusions about the extent of side effects in dependence of how these side effects are communicated. For this purpose you will be presented you with information on side effects for a drug and then asked some questions on your understanding of this information. Your data will be collected via a survey and your data will be kept anonymous.

The Max Planck Institute for Human Development is an institution that promotes scientific research. Our work adheres strictly to regulations governing protection of privacy. The information requested in the study will be kept confidential and archived and scientifically processed in accordance with the Data Privacy Act. Personal data will not be passed on to any third parties. The data will be used solely for research purposes and solely within the Max Planck Institute for Human Development in cooperation with the University of Hamburg. Personal contact data and experimental data will be stored separately from each other and handled with utmost discretion. Participation in the study is voluntary and you are able to end your participation at any time. You can revoke your consent to use your data at any time from that date forward.

The study will last approximately 15 minutes. You will be paid a compensation of 1 euro for your participation.

□ yes □ no I have read and accept the terms and conditions listed above and consent to participate in this study (*prerequisite for participating in the study*).

Name (in block letters): ______________________________

Date: ______________________________

Signature: ______________________________

Kollegium:
Ute Frevert
Gerd Gigerenzer
Ralph Hertwig
Ulman Lindenberger

Lentzeallee 94 D-14195 Berlin (Dahlem)
