# Supplementary material for: Alternative package leaflets improve people’s understanding of drug side effects—A randomized controlled exploratory survey
Source: PLoS One. 2018 Sep 13;13(9):e0203800. doi: 10.1371/journal.pone.0203800 (PMC6136776; doi:10.1371/journal.pone.0203800)
Supplement: S1 Fig — (PDF) [file pone.0203800.s003.pdf]

**S1 Fig. Format 1: Alternative package leaflet (intervention): Drug facts box (translation)**

**Side effects**

Like all medications, Suffia® can have side effects. Not all undesired symptoms are necessarily due to taking the drug. Undesired symptoms can also arise without taking the drug.

Occurrence of undesired symptoms over 5 years:

| **Undesired symptoms** | **Of 100 persons who take Suffia®:** | **Of 100 Persons who do NOT take Suffia®:** | **Undesired symptoms that are ascribed to Suffia® intake:** |
|---|---|---|---|
| Increased blood sugar levels | 16 of 100 | 13 of 100 | Taking Suffia® results in increased blood sugar levels in an additional 3 of 100 people. |
| Slow heart rate | 5 of 100 | 2 of 100 | Taking Suffia® results in slow heart rate in an additional 3 of 100 people. |
| Anemia | 4 of 100 | 4 of 100 | Taking Suffia® has no impact on anemia. |
| Depression | 9 of 100 | 12 of 100 | Taking Suffia® prevents depression in 3 of 100 people. |
