# Supplementary material for: Alternative package leaflets improve people’s understanding of drug side effects—A randomized controlled exploratory survey
Source: PLoS One. 2018 Sep 13;13(9):e0203800. doi: 10.1371/journal.pone.0203800 (PMC6136776; doi:10.1371/journal.pone.0203800)
Supplement: S2 Fig — (PDF) [file pone.0203800.s004.pdf]

S2 Fig. Format 2: Alternative package leaflet (intervention): Drug facts box with reading instruction (translation)

Side effects

Like all medications, Suffia® can have side effects. Not all undesired symptoms are necessarily due to taking the drug. Undesired symptoms can also arise without taking the drug.

	Occurrence of undesired symptoms over 5 years:		
**Undesired symptoms**	**Of 100 persons who take Suffia®:**	**Of 100 Persons who do NOT take Suffia®:**	**Undesired symptoms that are ascribed to Suffia® intake:**
Increased blood sugar levels	16 of 100	13 of 100	Taking Suffia® results in increased blood sugar levels in an additional 3 of 100 people.
Slow heart rate	5 of 100	2 of 100	Taking Suffia® results in slow heart rate in an additional 3 of 100 people.
Anemia	4 of 100	4 of 100	Taking Suffia® has no impact on anemia.
Depression	9 of 100	12 of 100	Taking Suffia® prevents depression in 3 of 100 people.

How to read this table: Of 100 people taking Suffia® over 5 years, 16 have increased blood sugar levels. Of 100 comparable people **not** taking Suffia®, 13 have increased blood sugar levels. Hence, 13 of 100 people taking Suffia® would have increased blood sugar levels even without taking the drug. Suffia® therefore causes an increase in blood sugar levels in 3 of 100 people.
