# Supplementary material for: Alternative package leaflets improve people’s understanding of drug side effects—A randomized controlled exploratory survey
Source: PLoS One. 2018 Sep 13;13(9):e0203800. doi: 10.1371/journal.pone.0203800 (PMC6136776; doi:10.1371/journal.pone.0203800)
Supplement: S3 Fig — (PDF) [file pone.0203800.s005.pdf]

S3 Fig. Format 3: Alternative package leaflet (intervention): Narrative with numbers (translation)

Side effects

Like all medicines, Suffia® can cause side effects. However, not all side effects are necessarily caused by the intake of Suffia®. Symptoms can also occur when Suffia® is not taken.

Symptoms occurring more often when taking Suffia® over 5 years:	
Increased blood sugar levels	16 of 100 people with Suffia® compared to 13 of 100 people without Suffia®.<br>In 3 of 100 people taking the drug Suffia® is the increase of blood sugar levels caused by the drug.
Slow heart rate	5 of 100 people with Suffia® compared to 2 of 100 people without Suffia®.<br>In 3 of 100 people taking the drug Suffia® is the slow heart rate caused by the drug.
Symptoms occurring less often when taking Suffia® over 5 years:	
Depression	9 of 100 people with Suffia® compared to 12 of 100 people without Suffia®.<br>In 3 of 100 people taking the drug Suffia® is depression prevented by the drug.
Symptoms occurring with comparable frequency when taking or NOT-taking Suffia® over 5 years:	
Anemia	4 of 100 people in each case.
