# Supplementary material for: Alternative package leaflets improve people’s understanding of drug side effects—A randomized controlled exploratory survey
Source: PLoS One. 2018 Sep 13;13(9):e0203800. doi: 10.1371/journal.pone.0203800 (PMC6136776; doi:10.1371/journal.pone.0203800)
Supplement: S4 Fig — (PDF) [file pone.0203800.s006.pdf]

S4 Fig. Format 4: Standard package leaflet (Control) (translation)

Side effects

Like all medications, Suffia® can have side effects, but not everybody will necessarily get them.

Possible side effects

Very common: affects more than 1 user in 10:

- Increased blood sugar levels

Common: affects up to 1 user in 10:

- Slow heart rate
- Anemia
- Depression
