# Supplementary material for: Alternative package leaflets improve people’s understanding of drug side effects—A randomized controlled exploratory survey
Source: PLoS One. 2018 Sep 13;13(9):e0203800. doi: 10.1371/journal.pone.0203800 (PMC6136776; doi:10.1371/journal.pone.0203800)
Supplement: S5 Fig — (PDF) [file pone.0203800.s007.pdf]

S5 Fig. Format 1: Drug facts box (Original language)

Nebenwirkungen

Wie alle Arzneimittel kann auch Suffia Nebenwirkungen haben. Dabei sind aber nicht alle unerwünschten Symptome auch zwangsläufig auf das Einnehmen von Suffia zurückzuführen. Unerwünschte Symptome können auch auftreten, wenn das Arzneimittel Suffia gar nicht eingenommen wird.

	Häufigkeit von unerwünschten Symptomen über 5 Jahre:		
**Unerwünschte Symptome**	**Von je 100 Personen, die Suffia® einnehmen:**	**Von je 100 Personen, die Suffia® NICHT einnehmen:**	**Unerwünschte Symptome, die auf die Einnahme von Suffia® zurückzuführen sind:**
Erhöhter Blutzucker	16 von 100	13 von 100	Das Einnehmen von Suffia® führt bei 3 zusätzlichen Personen von 100 zu einem erhöhten Blutzucker.
Langsamer Herzschlag	5 von 100	2 von 100	Suffia® führt bei 3 zusätzlichen Personen von 100 zu einem langsamen Herzschlag.
Blutarmut	4 von 100	4 von 100	Das Einnehmen von Suffia® hat keinen Einfluss auf Blutarmut.
Depression	9 von 100	12 von 100	Suffia® verhindert bei 3 von 100 Personen eine Depression.
