# Supplementary material for: Alternative package leaflets improve people’s understanding of drug side effects—A randomized controlled exploratory survey
Source: PLoS One. 2018 Sep 13;13(9):e0203800. doi: 10.1371/journal.pone.0203800 (PMC6136776; doi:10.1371/journal.pone.0203800)
Supplement: S7 Fig — (PDF) [file pone.0203800.s009.pdf]

S7 Fig. Format 3: Narratives with numbers (Original language)

Nebenwirkungen

Wie alle Arzneimittel kann auch Suffia Nebenwirkungen haben. Dabei sind aber nicht alle unerwünschten Symptome auch zwangsläufig auf das Einnehmen von Suffia zurückzuführen. Unerwünschte Symptome können auch auftreten, wenn das Arzneimittel Suffia gar nicht eingenommen wird.

Symptome, die häufiger unter der Einnahme von Suffia® über 5 Jahre auftreten:	
Erhöhter Blutzucker	16 von 100 Personen mit Suffia® im Vergleich zu 13 von 100 Personen ohne Suffia®.<br>Bei 3 von 100 Personen, die das Arzneimittel Suffia® einnehmen, ist das Arzneimittel die Ursache des erhöhten Blutzuckers.
Langsamer Herzschlag	5 von 100 Personen mit Suffia® im Vergleich zu 2 von 100 Personen ohne Suffia®.<br>Bei 3 von 100 Personen, die das Arzneimittel Suffia® einnehmen, ist das Arzneimittel die Ursache des langsamen Herzschlags.
Symptome, die seltener unter der Einnahme von Suffia® über 5 Jahre auftreten:	
Depression	9 von 100 Personen mit Suffia® im Vergleich zu 12 von 100 Personen ohne Suffia®.<br>Bei 3 von 100 Personen, die Suffia® einnehmen, wird eine Depression durch die Einnahme des Arzneimittels Suffia® verhindert.
Symptome, die mit vergleichbarer Häufigkeit unter der Einnahme und unter der NICHT-Einnahme von Suffia® über 5 Jahre berichtet wurden:	
Blutarmut	Je 4 von 100 Personen.
