# Supplementary material for: Alternative package leaflets improve people’s understanding of drug side effects—A randomized controlled exploratory survey
Source: PLoS One. 2018 Sep 13;13(9):e0203800. doi: 10.1371/journal.pone.0203800 (PMC6136776; doi:10.1371/journal.pone.0203800)
Supplement: S8 Fig — (PDF) [file pone.0203800.s010.pdf]

S8 Fig. Format 4: Standard package leaflet (Original language)

Nebenwirkungen

Wie alle Arzneimittel kann auch Suffia Nebenwirkungen haben, die aber nicht bei jedem auftreten müssen.

Mögliche Nebenwirkungen:

<u>Sehr häufig: kann mehr als 1 von 10 Behandelten betreffen</u>

- Erhöhter Blutzucker

<u>Häufig: kann bis zu 1 von 10 Behandelten betreffen</u>

- Langsamer Herzschlag
- Depression
- Blutarmut
