# Supplementary material for: Alternative package leaflets improve people’s understanding of drug side effects—A randomized controlled exploratory survey
Source: PLoS One. 2018 Sep 13;13(9):e0203800. doi: 10.1371/journal.pone.0203800 (PMC6136776; doi:10.1371/journal.pone.0203800)
Supplement: S1 Table — (PDF) [file pone.0203800.s011.pdf]

S1 Table. Excerpt from Barron et al.: The proportion of side-effects on beta-blocker that are caused by being on beta-blocker

Side effect	Beta-blocker			Placebo			p-value	Number of patients (from 100 presenting with a symptom) where Beta-blockers are not causative
	%	n (SE)	n (total)	%	n (SE)	n (total)		
Hyperglycemia	16.1	300	1858	13.4	213	1585	0.026	83
Bradycardia	4.9	387	7836	1.6	122	7547	<0.001	33
Anemia	3.5	37	1067	3.6	38	1061	0.887	
Depression	8.5	119	1398	11.5	159	1383	0.009	
