# Supplementary material for: Alternative package leaflets improve people’s understanding of drug side effects—A randomized controlled exploratory survey
Source: PLoS One. 2018 Sep 13;13(9):e0203800. doi: 10.1371/journal.pone.0203800 (PMC6136776; doi:10.1371/journal.pone.0203800)
Supplement: S2 Table — (PDF) [file pone.0203800.s012.pdf]

S2 Table. Distribution of participants' responses on the general occurrence of side effects during drug intake for each format

Item	Drug facts Box	Drug facts box with reading instruction	Narrative with numbers	Standard package leaflet
	Mean (median; Standard Deviation)			
Hyperglycemia	13.13 (16.00; SD = 5.65)	10.36 (16.00; SD = 6.59)	10.27 (16.00; SD = 6.47)	13.09 (10.00; SD = 14.14)
Bradycardia	4.67 (5.00; SD = 1.27)	4.26 (5.00; SD = 1.21)	4.11 (5.00; SD = 1.01)	8.63 (10.00; SD = 9.18)
Anemia	3.03 (4.00; SD = 1.72)	2.31 (4.00; SD = 2.04)	3.93 (4.00; SD = .76)	8.43 (10.00; SD = 8.62)
Depression	7.62 (9.00; SD = 3.17)	5.80 (9.00; SD = 3.94)	7.48 (9.00; SD = 9.39)	8.73 (10.00; SD = 9.35)
