# Supplementary material for: Alternative package leaflets improve people’s understanding of drug side effects—A randomized controlled exploratory survey
Source: PLoS One. 2018 Sep 13;13(9):e0203800. doi: 10.1371/journal.pone.0203800 (PMC6136776; doi:10.1371/journal.pone.0203800)
Supplement: S4 Table — (PDF) [file pone.0203800.s014.pdf]

S4 Table. Effect of the order of the question of general occurrence on the proportion of participants' correct responses in dependence on whether participants first received the question on general occurrence and then the question on causality or vice versa

* *p value cannot be calculated because no participant provided a correct response to that question*

Item	Drug facts Box	Drug facts box with reading instruction	Narrative with numbers	Standard package leaflet
	Pearson Chi Square Test of order effects on responses (Contingency Coefficient)			
Hyperglycemia	p = .08 (r_φ = .19)	p = 1.00 (r_φ = .02)	p = .33 (r_φ = .11)	*
Bradycardia	p = .08 (r_φ = .19)	p = .83 (r_φ = .04)	p = .43 (r_φ = .09)	p = 1.00 (r_φ = .01)
Anemia	p = .09 (r_φ = .18)	p = .30 (r_φ = .11)	p = .12 (r_φ = .17)	p = .51 (r_φ = .12)
Depression	p = .04 (r_φ = .22)	p = .53 (r_φ = .08)	p = .55 (r_φ = .07)	p = .76 (r_φ = .03)
